# Signaling mechanism by the *Staphylococcus aureus* two-component system LytSR: role of acetyl phosphate in bypassing the cell membrane electrical potential sensor LytS

**DOI:** 10.12688/f1000research.6213.2

**Published:** 2016-03-22

**Authors:** Kevin Patel, Dasantila Golemi-Kotra

**Affiliations:** 1 Department of Chemistry, York University, Toronto, Toronto, Ontario, M3J 1P3, Canada; 2 Department of Biology, York University, Toronto, Toronto, Ontario, M3J 1P3, Canada

**Keywords:** LytSR, Histidine kinase, Response Regulator, Phosphotransfer, Staphylococcus Aureus

## Abstract

The two-component system LytSR has been linked to the signal transduction of cell membrane electrical potential perturbation and is involved in the adaptation of *Staphylococcus aureus* to cationic antimicrobial peptides. It consists of a membrane-bound histidine kinase, LytS, which belongs to the family of multiple transmembrane-spanning domains receptors, and a response regulator, LytR, which belongs to the novel family of non-helix-turn-helix DNA-binding domain proteins. LytR regulates the expression of *cidABC* and *lrgAB* operons, the gene products of which are involved in programmed cell death and lysis. *In* *vivo* studies have demonstrated involvement of two overlapping regulatory networks in regulating the *lrg*AB operon, both depending on LytR. One regulatory network responds to glucose metabolism and the other responds to changes in the cell membrane potential. Herein, we show that LytS has autokinase activity and can catalyze a fast phosphotransfer reaction, with 50% of its phosphoryl group lost within 1 minute of incubation with LytR. LytS has also phosphatase activity. Notably, LytR undergoes phosphorylation by acetyl phosphate at a rate that is 2-fold faster than the phosphorylation by LytS. This observation is significant in lieu of the *in vivo* observations that regulation of the *lrgAB* operon is LytR-dependent in the presence of excess glucose in the medium. The latter condition does not lead to perturbation of the cell membrane potential but rather to the accumulation of acetate in the cell. Our study provides insights into the molecular basis for regulation of *lrgAB* in a LytR-dependent manner under conditions that do not involve sensing by LytS.

## Summary statement

The molecular basis of signal transduction by LytSR is unknown. We show that LytS has kinase and phosphatase activity. LytR undergoes rapid phosphorylation by acetyl phosphate. Activity of LytR is regulated either through LytS or acetyl phosphate. LytSR is at the interface of two regulatory pathways that respond to excess glucose metabolism and cell membrane electrical potential, respectively.

## Introduction

The two-component system LytSR of *Staphylococcus aureus* is reported to function as a sense-response system for detecting subtle changes in the electrical potential of the cell membrane. It is also involved in adaptation of *S. aureus* to cationic antimicrobial peptides (CAMPs) ^1– 3^. CAMPs are bactericidal agents released by the host innate immune system during colonization by *S. aureus* ^4, 5^. Their mechanism of action is believed to involve perturbation of cell membranes which, in turn, alters the electrical potential of the cell membrane ^3, 6^. The function of LytSR in the cell is reported as a regulator of cell wall lysis during programmed cell death and biofilm formation ^7– 12^.

A typical two component system (TCS) consists of a membrane bound histidine kinase (HK) which intercepts an environmental cue and through an act of auto-phosphorylation transduces the signal intracellularly. The response to the cue is mediated through a phosphotransfer process whereby a second protein, in response to a regulator protein, receives the phosphoryl group from the cognate histidine kinase (HK) at a conserved aspartate residue. The response regulator (RR) protein is often a transcription factor and in some cases an enzyme ^13^. LytSR is comprised of the membrane-bound sensor HK LytS and the RR protein LytR. LytS belongs to a family of bacterial receptor proteins that contain five transmembrane-spanning domains. LytR is a transcription factor that falls into a novel family of proteins that contain non-helix-turn-helix DNA-binding domains, known as LytTRs ^14, 15^.

LytR regulates *lrgAB* operon in response to alterations in the electrical potential of the cell membrane ^16^. The *lrgAB* operon together with *cidABC* operon are involved in the control of programmed cell death and lysis during biofilm formation ^12, 17, 18^; the gene products of the *cid* operon enhance murein hydrolysis activity and antibiotic tolerance ^19^ while the *lrg* genes inhibit these processes ^9^. Interestingly, both operons were also shown to be induced by carbohydrate metabolism ^16^ and proposed to be regulated through a *cidR*-dependent signaling pathway ^1^. The *cidR* gene encodes a LysR-type transcription factor which has been proposed to be activated by accumulation of acetate during metabolism of excess glucose by *S. aureus* at logarithmic growth ^20, 21^. Recent work by Sharma-Kuinkel *et al*. ^2^ demonstrated that *lrgAB* is instead regulated only through LytR, either in response to carbohydrate metabolism (e.g. excess of glucose) or as a result of disruption of cell membrane electrical potential. However, the molecular basis for this observation remained obscure.

To examine the signal transduction mechanism of LytSR and probe its involvement in the regulation of *lrgAB* in response to carbohydrate metabolism as a result of a phosphorylation-induced activation of LytR by acetyl phosphate, we cloned and purified LytS and LytR and investigated the autokinase activity of LytS, the kinetics of the phosphotransfer between LytS and LytR, and the kinetics of phosphorylation of LytR by acetyl phosphate. Our study shows that LytSR is capable of mediating signaling either through LytS in response to cell membrane electrical potential or through LytR in response to carbohydrate metabolism. Furthermore, phosphorylation-induced activation of LytR by either LytS or acetyl phosphate is likely to involve dimerization of LytR at the receiver domain.

## Materials and methods

### Chemical reagents and materials

Chemicals and antibiotics were purchased from Sigma (Oakville, Canada) or Thermo-Fisher (Whitby, Canada), unless otherwise stated. Chromatography media and columns were purchased from GE Healthcare (Quebec, Canada). Growth media were purchased from Fisher. *Escherichia coli* strains, NovaBlue and BL21 (DE3), and cloning and expression plasmids were purchased from EMD4 Biosciences (New Jersey, USA). The pGEX-4T vector was purchased from GE Healthcare (Quebec, Canada). Restriction enzymes were obtained from New England Biolabs (Pickering, Canada) or Thermo-Fisher. The [γ- ^32^P] Adenosine triphosphate (ATP) was purchased from Perkin Elmer LAS Canada Inc. (Toronto, Canada) or GE Healthcare. The Proteo Extract All-in-One Trypsin Digestion Kit was purchased from EMD4 Bioscience. The genome of the *S. aureus* strain Mu50 was obtained from Cedarlane (Burlington, Canada).

### Cloning of the *lytS* gene that encodes the cytoplasmic domain into an over-expression plasmid

The gene sequence of *lytS* (SAV0260, as per gene numbering in *S. aureus* Mu50 strain) encoding the cytoplasmic region of the protein (amino acid residues 355–584) was amplified from *S. aureus* Mu50 genome using the primers: Dir 5'- *CACC*GCAGAAGGATTGGCAAAT-3' and Rev 5'-TTATTCCTCCTCTTG TCTTT CA-3'. To enable directional cloning, the forward primer contained a specific 4 base pair (bp) sequence (italicized) at the 5' end of the primer. The 701 bp *lytS* gene was amplified using Phusion High-Fidelity DNA polymerase with the following PCR conditions: initial denaturation at 98°C for 30 s, followed by 30 cycles at 98°C at 10 s, annealing at 61°C for 20 s, extension at 72°C for 20 s and final extension at 72°C for 10 min. The blunt ended amplicon was then ligated to pET151/D-TOPO vector using the Champion™ pET Directional TOPO ^®^ Expression Kit. The ligated *pET151/D-TOPO::lytS* construct was used to transform *E. coli* NovaBlue cells for further amplification. The correct sequence of *lytS* was confirmed by DNA sequencing (The Centre for Applied Genomics, the Hospital for Sick Children, Toronto, Canada. The *pET151/D-TOPO::lytS* plasmid was used to transform the *E. coli* BL21 (DE3) to facilitate protein expression. This cloning strategy resulted in introduction of an NH _2_-terminous 6xHis tag upstream of *lytS*.

### Production and purification of His-LytS

*E. coli* BL21(DE3) cells carrying the construct *pET151/D-TOPO::lytS* were used to inoculate 5 mL of Luria Bertani (LB) medium supplemented with 100 µg/mL final concentration of ampicillin and allowed to grow overnight at 37°C by shaking. An aliquot of 1 mL of the overnight cell culture was used to inoculate 1 L of Terrific Broth (TB) medium supplemented with 100 µg/mL of ampicillin. Cells were allowed to grow at 37°C by shaking at 200 rpm until the cell culture reached an optical density of 0.6 to 0.8 absorbance units at 600 nm (OD _600nm_). At this point, the cell culture was cooled to 4°C and protein production initiated by adding isopropyl β‐D‐1‐thiogalactopyranoside (IPTG) at a final concentration of 0.1 mM. Cell culture was shaken at 200 rpm at 18°C for 12 hrs. Cells were harvested by centrifugation at 3,300 × *g* for 20 min.

For isolation of His-LytS, the cell pellet was resuspended in 50 mM sodium phosphate pH 7.5, supplemented with 300 mM NaCl and 10 mM Imidazole. The cellular content was liberated by sonication while cooling on ice for 10 min (10s on/15s off) and cell debris was removed by centrifugation at 18,000 × *g* for 1 h at 4°C. Purification of His-LytS was carried out by loading the supernatant onto a self-assembled affinity column (Generon, UK) packed with 8 mL of Ni-NTA resin. All purification steps were carried out at 4°C using the AktÄ purifier 10 (GE Healthcare). The unbound protein was removed by washing with buffer for three column volumes (CV). The protein of interest was eluted using a step gradient of 10%, 40%, 70% and 100% of 300 mM imidazole in 50 mM sodium phosphate pH 7.5 buffer, supplemented with 300 mM NaCl, at a flow rate of 1.5 mL/min in three CV. Fractions of 5 mL containing the protein were concentrated using Amicon Ultra-10K concentrator (Millipore) followed by dialysis into the storage buffer: 50 mM Tris pH 7.5, supplemented with 150 mM NaCl and 5 mM MgCl _2_. The homogeneity of protein was assessed by loading samples onto a 15% sodium dodecyl sulphate-polyacrimide gel electrophoresis (SDS-PAGE) apparatus and staining the gel with Coomassie blue.

### Cloning of the full length *lytR* gene into an over-expression plasmid

The gene sequence of *lytR* (SAV0261) was amplified from the *S. aureus* Mu50 genome using the primers: Dir 5'-GGAATTC *CATATG*AAAGCATTAATCATAGATGATG-3' and Rev 5'-CGG *AATTC*TTAT TAAAGTAATCCTA TCGACG-3'. The primers were designed to introduce the *NdeI* and *EcoRI* restriction sites (italicized sequences) at 5' and 3' of *lytR*, respectively. Amplification of the 741 bp *lytR* gene was carried out using Phusion ^®^ High-Fidelity DNA polymerase following these conditions: initial denaturation at 98°C for 30 s, followed by 30 cycles at 98°C at 10 s, annealing at 62°C for 20 s, extension at 72°C for 20 s and final extension at 72°C for 10 min. The resulting amplicon was purified and together with the host vector, pET26b, was digested with *Nde*I and *EcoR*I. The digestion products were gel purified from 1% agarose gel using the QIAquick Gel Extraction Kit (Qiagen) and subjected to ligation using T4 DNA ligase (NOVAGEN). The subsequent construct was referred to as *pET26b::lytR* and was used to transform *E. coli* NovaBlue cells for further amplification. The correct sequence of the *lytR* gene was confirmed by DNA sequencing (The Centre for Applied Genomics, the Hospital for Sick Children, Toronto, Canada). The *pET26b::lytR* plasmid was used to transform the *E. coli* BL21 (DE3) expression cells. This cloning strategy resulted in introduction of no tags or additional amino acids to LytR.

### Cloning of the lytR gene that encodes for the receiver domain (lytR ^N^) into an over-expression plasmid

To clone the receiver domain of the LytR protein, LytR ^N^ (residues 1-134), a stop codon after the 134 ^th^ amino acid residue was introduced using the QuikChange ^®^ Site-Directed Mutagenesis method (Thermo Fisher). The process of site directed mutagenesis was carried out using the *Pfu Turbo* ^®^ DNA polymerase and the following mutagenic primers: 5'-GCGAATGATATGTCG *TAG*AATTTTGATCAAAGC-3' and 3'-GCTTTGATCAAAATT *CTA* CGACATATCATTCGC-5' (mutated nucleotides are italicized). The construct *pET26b::lytR* was used as the template. The amplified mutagenic construct *, pET26b::lytR ^N^* was treated with the restriction endonuclease *DpnI* and used to transform *E. coli* NovaBlue cells. Successful insertion of the stop codon was confirmed by DNA sequencing (The Centre for Applied Genomics, the Hospital for Sick Children, Toronto, Canada) and the *pET26b::lytR* ^*N*^ vector was used to transform *E. coli* BL21 (DE3).

### Cloning of lytR fused to the COOH-terminus of the Glutathione S-Transferase protein (GST) and construction of the Asp53Ala mutant of GST-LytR

The following primer set was used to clone the full length *lytR* into pGEX-4T-1 to enable fusion of the protein to the C-terminal of GST, Dir 5'-AGTCG *GGATCC*ATGAAAGCATTAATCATA GATG-3' and Rev 5'-CG *GAATTC*TTATTAAAGTAATCCTATCG ACG-3'. The primers were designed to introduce *BamHI* and *EcoRI* restriction sites (italicized sequences) at 5' and 3' of *lytR* ends respectively, to enable cloning.

The Asp-53 residue of LytR was mutated to an Ala residue using QuikChange ^®^ Site-Directed Mutagenesis (Thermo Fisher). Briefly, the process of site directed mutagenesis was carried out using the *Pfu Turbo* ^®^ DNA polymerase with the designed mutagenic primers: 5'-AC ATTATATTTTTA *GCT*GTCAATTTAATGG-3' and 3'-CCATTAAATTGAC *AGC*TAAAA AT ATAATGTC- 5' (mutated nucleotides are italicized). The construct *pGEX-4T1::lytR* was used as a template. The amplified mutagenic construct, referred to as *pGEX-4T::lytR*Asp53Ala, was treated with the restriction endonuclease *DpnI* and used to transform *E. coli* NovaBlue. Successful mutation of Asp to Ala was confirmed by DNA sequencing (The Centre for Applied Genomics, the Hospital for Sick Children, Toronto, Canada). The *pGEX-4T::lytR*Asp53Ala plasmid was used to transform the *E. coli* BL21 (DE3).

### Production and purification of LytR

In general, *E. coli* BL21 (DE3) carrying the appropriate plasmid was used to inoculate 5 mL of LB in the presence of 50 µg/mL of kanamycin. An aliquot of 1 mL of the overnight cell culture was used to inoculate 1 L of TB supplemented with 50 µg/mL of kanamycin. The cells were allowed to grow to OD _600nm_ = 0.6–0.8 with shaking at 200 rpm at 37°C. Once desired growth was achieved the media was cooled to 4°C and protein production was induced by adding IPTG to a final concentration of 0.1 mM. Cell culture was allowed to shake for an additional 12 h at 18°C. Cells were harvested by centrifugation at 3,300 × *g* for 20 min.

To isolate LytR the method of protein precipitation by ammonium sulfate was employed. All purifications steps were carried out at 4°C. Cell pellet was suspended in 1:10 (w/v) of 50 mM Tris, pH 8.0, 100 mM NaCl, 5 mM MgCl _2_ supplemented with 10% glycerol. The cellular content was liberated by sonication and cell debris was removed by centrifugation at 18,000 × *g* for 1 h at 4°C. Total volume of supernatant was adjusted to be 50 mL (for 5 g cell) and 2.67 g of ammonium sulfate was added gently while stirring, to achieve saturation of 10%. The solution was incubated while stirring at 4°C for 30 min. The precipitated protein was collected by centrifugation at 3,300 × *g* for 5 min. The protein pellet was dissolved in 10 mL of 50 mM Tris, pH 8.0, 100 mM NaCl, 5 mM MgCl _2_ supplemented with 10% glycerol. The purity of the protein was evaluated by 15% SDS-PAGE stained with Coomassie blue. The protein solution was dialysed to remove ammonium sulfate.

### Production and purification of LytR ^N^

*E. coli* BL21 (DE3) harboring *pET26b::lytR ^N^* was used to inoculate 5 mL of LB in the presence of 50 µg/mL kanamycin. An aliquot of 1 mL of the overnight cell culture was used to inoculate 1 L of TB supplemented with 50 µg/mL of kanamycin. The cells were allowed to grow to an OD _600nm_ = 0.6–0.8 while shaking at 200 rpm at 37°C. The cell culture was cooled to 4°C and protein production initiated by addition of 0.5 mM IPTG. The cell culture was allowed to shake for an additional 12 h at 25°C for. Cells were harvested by centrifugation at 3,300 × *g* for 20 min.

To isolate LytR ^N^, cell pellet was suspended in 1:10 (w/v) of 20 mM Tris pH 7.5, supplemented with 5 mM MgCl _2_. The cellular content was liberated by sonication and cell debris was removed by centrifugation at 18,000 × *g* for 1 h at 4°C. The supernatant was loaded onto a DEAE-Sepharose™ column (GE Healthcare) and mounted into the AktÄ purifier 10. The protein was eluted over ten column volumes in a linear gradient of 20–500 mM Tris (pH 7.5) (supplemented with 5 mM MgCl _2_) at a flow rate of 3 mL/min. Elution fractions containing protein were pooled together and concentrated by centrifugation using Amicon Ultra-3K concentrator (Millipore) to a final volume of 5 mL. The protein was loaded onto a HiPrep 26/60 Sephacryl S-200 HR gel-filtration column (GE Healthcare). The homogeneity of the protein was determined using 18% SDS-PAGE.

### Production and purification of GST-LytR and GST-LytR-Asp53Ala

*E. coli* BL21 (DE3) carrying the desired plasmid were used to inoculate 5 mL of LB in the presence of 100 µg/mL ampicillin. An aliquot of 1 mL of the overnight grown seed culture was used to inoculate 1 L of TB supplemented with 100 µg/mL of ampicillin. Cells were allowed to grow to an OD _600nm_ = 0.6–0.8 at 37°C while shaking at 200 rpm. Then the cell culture was cooled to 4°C and protein production was initiated by addition of 0.5 mM IPTG and incubated further at 18°C for 12 h. The cells were harvested by centrifugation at 3,300 × *g* for 20 min.

For purification of the wild type or mutant GST-LytR protein, the cell pellet was suspended in 1:10 (w/v) of 50 mM Tris pH 7.5, supplemented with 100 mM NaCl and 5 mM MgCl _2_. The cellular content was liberated by sonication and cell debris was removed by centrifugation at 18,000 × *g* for 1 hour at 4°C. Purification was carried out by loading the supernatant onto a self-assembled affinity column packed with 5 mL of GST affinity resin (Generon). The protein of interest was eluted at a flow rate of 1.5 mL/min with 10 mM reduced glutathione in 50 mM Tris-HCl pH 8.0 buffer over three CV. Fractions containing the protein were concentrated using Amicon Ultra-10K concentrator (Millipore) followed by dialysis to exchange the buffer into 50 mM Tris pH 7.5, supplemented with 100 mM NaCl and 5 mM MgCl _2_. The homogeneity of protein was assessed by 12.5% SDS-PAGE stained with Coomassie blue.

The identities of the all proteins isolated in this study were confirmed by cutting the protein band from the SDS-PAGE gel, subjecting this to trypsin digestion and submitting the digest for mass spectrometry analysis. The molecular mass of the purified proteins was determined by electrospray ionization mass spectrometry (ESI-MS). All the mass spectrometry analyses were done at the Advanced Protein Technology Centre, Hospital for Sick Children (Toronto, Canada).

### Assessment of the autokinase activity of LytS

His-LytS at 5 µM was equilibrated in phosphorylation buffer (PB: 50 mM Tris, pH 7.4, 50 mM KCl, 5 mM MgCl _2_) supplemented with 10 mM CaCl _2_. The phosphorylation reaction was initiated by the addition of [γ- ^32^P]-ATP (10 Ci/mmol) mixed with cold ATP to prepare reaction mixtures at different ATP final concentrations. Samples were incubated at room temperature. The reaction was stopped at different time intervals by adding 5 × SDS sample buffer (125 mM Tris, pH 6.8, 2.5% SDS, 25% glycerol, 100 mM dithiothretiol (DTT), 0.0025% bromophenol blue). Samples were loaded onto a 15% SDS-PAGE. The SDS-PAGE gels were washed in water containing 2% (v/v) glycerol and were exposed to a phosphor screen (GE Healthcare) overnight and imaged using a Typhoon Trio ^+^ imager (GE Healthcare). The radioactive gels were stained by Coomassie blue dye to analyze protein content of the samples.

Each time-dependent phosphorylation experiment was repeated twice. The progress of the reaction was assessed by analyzing the phosphor-image of the radioactive gels using NIH ImageJ software (Version 1.45s) (freely available at http://imagej.nih.gov/ij/download.html). The progress curves were fitted to the first-order rate Equation 1, where I is the band intensity quantified by NIH ImageJ, *k _obs_* is the observed rate constant, t is the incubation time and A is the proportionality constant relating intensity with concentration of phosphorylated His-LytS.

*I* = *A*×{1 – exp(– *kobs* × *t*)}      (1)

The data were fitted using Erithacus GraFit software (version 5.0.10) (available at http://www.erithacus.com/grafit/Software_Updates.htm). The observed phosphorylation rate constant was calculated for each ATP concentration and was plotted against each ATP concentration to determine the rate constant of autophosphorylation of LytS using the equation (2), where *k*_obs_ is the observed rate constant measured at each ATP concentration, *k* is the autophosphorylation rate constant for LytS, *K*_s_, is the dissociation constant of ATP and [S] the ATP concentration.

$$kobs\;=\;k\times \frac{\left[S\right]}{Ks+[S]}\;\;\;\;\;(2)$$

To study the effect of salt ions on the phosphorylation of His-LytS, we looked at the effect of K ^+^ and Ca ^2+^. His-LytS at 3 µM was equilibrated in the phosphorylation buffer (PB: 50 mM Tris, pH 7.4, 5 mM MgCl _2_) with varying concentrations of either KCl or CaCl _2_. The phosphorylation reaction was initiated by the addition of [γ- ^32^P]-ATP (10 Ci/mmol) to a final concentration of 20 µM. The reaction was incubated for 90 min at RT then quenched by adding 5 × SDS sample buffer. Samples were loaded onto a 15% SDS-PAGE. The radioactive gels were washed in water containing 2% (v/v) glycerol and gels were exposed to a phosphor screen (GE Healthcare) overnight. The screen was imaged using a Typhoon Trio ^+^ imager (GE Healthcare).

To investigate the stability of the phosphorylated state of LytS, His-LytS at 10 µM was allowed to undergo autophosphorylation for 90 min by adding [γ- ^32^P]-ATP (10 Ci/mmol) to a final concentration of 20 µM. Excess [γ- ^32^P]-ATP was removed by desalting using the Zeba Spin Desalting column (Pierce, Thermo Scientific) equilibrated with PB. The reaction mixture was further incubated at room temperature and aliquots were removed at different time intervals and quenched by adding 5 × SDS sample buffer. Samples were loaded onto 15% SDS-PAGE and the gel was analyzed as described above.

### Phosphorylation of LytR by LytS and small molecule phosphate donors

The ability of LytS to phosphorylate LytR was examined as described earlier ^22^. Briefly, His-LytS at 15 µM was phosphorylated for 90 min. Excess [γ- ^32^P]-ATP was removed by desalting using the Zeba Spin Desalting column which was equilibrated with PB. Phosphorylated His-LytS (4 µM) was incubated with GST-LytR (10 µM) in the PB buffer at room temperature. Aliquots of 10 µL were removed at different time intervals and quenched by adding 10 µL of 5 × SDS-PAGE sample buffer. Samples were loaded onto a 15% SDS-PAGE. The radioactive gels were washed with water containing 2% (v/v) glycerol and gels were exposed to phosphor screen (GE Healthcare) overnight and imaged using a Typhoon Trio ^+^ imager (GE Healthcare). The radioactive gels were stained by Coomassie blue dye. The phosphor images of the radioactive gels were quantified using NIH ImageJ software (Version 1.45s). Similar experiments were carried out using GST-LytR-D53A mutant and LytR ^N^.

To investigate phosphorylation of LytR by small molecule phosphate donors, lithium potassium acetyl phosphate was used as described earlier ^22^. Briefly, LytR at 10 µM or LytR ^N^ at 20 µM was equilibrated in PB20 buffer (PB: 50 mM Tris, pH 7.4, 50 mM KCl, 20 mM MgCl _2_) and phosphorylation was initiated by addition of lithium potassium acetyl phosphate to a final concentration of 50 mM. The reaction mixture was incubated at 37°C and 15 µL aliquots were removed and quenched by adding 5 × SDS sample buffer. The phosphorylated species were separated from unphosphorylated species using a 15% SDS-PAGE containing Acrylamide-pendant Phos-tag™ AAL-107 at 50 µmol/L (Wako chemical USA, inc., Cedarlane) ^23^. The gels were stained by Coomassie blue dye. Band intensities of the phosphorylated species were quantified using NIH ImageJ and plotted against incubation time. These data were fitted to Equation 1 using Erithacus GraFit software (version 5.0.10) to determine the phosphorylation rate constants of LytR and LytR ^N^ by acetyl phosphate.

To investigate the effect of phosphorylation on oligomerization state of LytR or LytR ^N^, each protein was phosphorylated by acetyl phosphate as described above. Unphosphorylated and phosphorylated samples of LytR ^N^ (10 µM, 20 µM and 40 µM) were loaded onto a 10% native-PAGE. The internal temperature of the buffer during the gel electrophoresis was maintained at 4°C. The gels were stained with Coomassie blue to visualize the protein bands. We analyzed phosphorylated and unphosphorylated LytR ^N^ (80 µM) also by size exclusion chromatography TSKgel G2000SW _XL_ (7.8 × 300mm, 5 µm, TOSOH Biosciences LLC) on HPLC.

### Phosphatase activity of LytS

To assess the phosphatase activity of LytS, LytR ^N^ was phosphorylated by acetyl phosphate as described above. Excess acetyl phosphate was removed by the desalting column. LytR ^N^-P (80 µM) was incubated with 5 µM of LytS at different time intervals, in the absence or presence of 200 µM ATP, at 37°C. Samples were quenched with native-PAGE loading buffer and loaded onto a 15% native-PAGE. Native-PAGE was stained with Coomassie blue to visualize the protein bands which were quantified by NIH ImageJ.

### Circular dichroism spectroscopy

Far UV circular dichroism (CD) spectra (200–260 nm) of LytR and LytR ^N^ were recorded using a Jasco J-180 instrument at 20°C _._ The buffer composition in these experiments was 20 mM Tris, 5 mM MgCl _2_ pH 8.0. Two spectra scans were averaged for the sample and the buffer. Later, buffer contribution was subtracted from each protein spectrum. To assess the thermal stability of LytR and LytR ^N^, thermal melting of each protein was recorded by monitoring the change in the CD signal at 222 nm by ramping up the temperature from 20°C to 90°C at a rate of 3°C/min. Typically for these experiments, 20 µM of the respective protein was buffer exchanged into 10 mM sodium phosphate buffer at pH 7.4.

## Results

### Modular architectures of LytS and LytR

**Figure 1.**
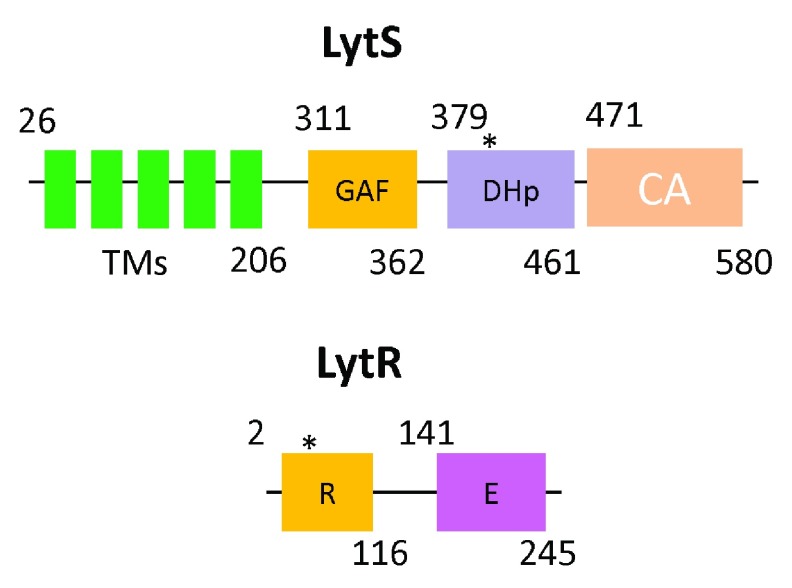
Domain organizations of LytS (P60612) and LytR (P60609) as predicted by InterPro (EMBL-EBI) (“TM” stands for transmembrane regions; “*” denotes the phosphorylation sites, His-390 in LytS and Asp-53 in LytR; “R” stands for receiver domain; “E”, stands for the effector domain).

The predicted domain architectures of LytS and LytR are shown in Figure 1. LytS belongs to the family of LytS-YhcK multi-transmembrane domain bacterial receptors which carry at their intracellular C-terminal a GAF (cyclic Guanosine Monophosphate-specific phosphor-diesterases, Adenylyl cyclases and the FhlA proteins ^24^) domain, and a kinase domain ^25^. The amino acid analysis of LytS by InterPro (EMBL-EBI; available online at http://www.ebi.ac.uk/interpro/) predicted that this protein has five transmembrane regions (TMs), an extracellular NH _2_-terminus, and an intracellular GAF domain and a kinase domain. The intracellular kinase domain harbors the dimerization and histidine phosphotransfer (DHp) domain and the catalytic and ATP-binding (CA) domain.

LytR consists of two domains, the conserved N-terminal receiver domain (LytR ^N^) and the variable C-terminal DNA-binding domain referred to as the effector domain (LytR ^C^). In general, the receiver domain of the RRs harbors a conserved Asp residue that undergoes a reversible phosphorylation by the cognate HK ^26, 27^. The sequence analysis of LytR reveals that the receiver domain is homologous to the receiver domains of the OmpR/NarL protein families. Based on the sequence alignments with these RRs, we determined the phosphorylation site in LytR to be Asp-53. Sequence analysis of LytR also indicates that the DNA-binding domain is homologous to that found in the novel family of non-helix-turn-helix DNA-binding domains, known as LytTR ^14, 15^. This family of proteins consists of AlgR and AgrA transcription factors ^15^ which are involved in regulation of important virulence factors in pathogenic bacteria ^28^. These groups of effector domains are unique in their ability to bind to DNA and account for ~2.7% of all prokaryotic RRs ^28^.

### Expression and purification of His-LytS and LytR proteins

The cloning strategy of *lytS* aimed to clone the cytoplasmic region of LytS spanning the amino acids 355-584, which harbors the DHp and CA domains. The cytoplasmic domain of LytS was fused at the C-terminus of a six-histidine tag (calculated molecular mass of the His-LytS is 28,359 D). The (His) _6_-tag aided purification of LytS to homogeneity as assessed by SDS-PAGE.

Cloning of *lytR* (246 amino acids) led to the expression of LytR without tags or additional amino acids (calculated molecular mass 28,221 D). Expression of LytR was good but its purification was challenging. Conventional chromatographic methods failed, and it was evident that the actual isoelectric point (pI) was different from the theoretical one (pI ~5.68). We resorted to the use of ammonium sulfate precipitation to purify the protein. The protein precipitated out at 10% ammonium sulfate and the purity was assessed to be 80%. The protein was prone to aggregation at concentrations higher than 3 mg/mL. To facilitate purification and solubility of the protein, we cloned LytR fused to the COOH-terminus of GST. GST-LytR was purified to homogeneity by affinity chromatography.

Cloning of the receiver domain of LytR, LytR ^N^, spanning amino acids 1-134, led to the expression of a soluble protein with a molecular mass of 15,028 D without additional amino acids or tags. The protein was expressed at a higher level than LytR or GST-LytR. Moreover the protein was purified by conventional chromatographic methods based on the theoretical value of pI ~4.44. The protein was very stable and soluble at concentrations as high as 10 mg/mL.

The thermal melting points of LytR and LytR ^N^ were measured to be 55°C and 70°C, respectively. The 15 degree difference in the thermal stabilities between these two proteins is an indication that the effector domain may destabilize the N-terminal domain in the context of the full-length protein.

### Kinetics of the autokinase activity of His-LytS

**Figure 2.**
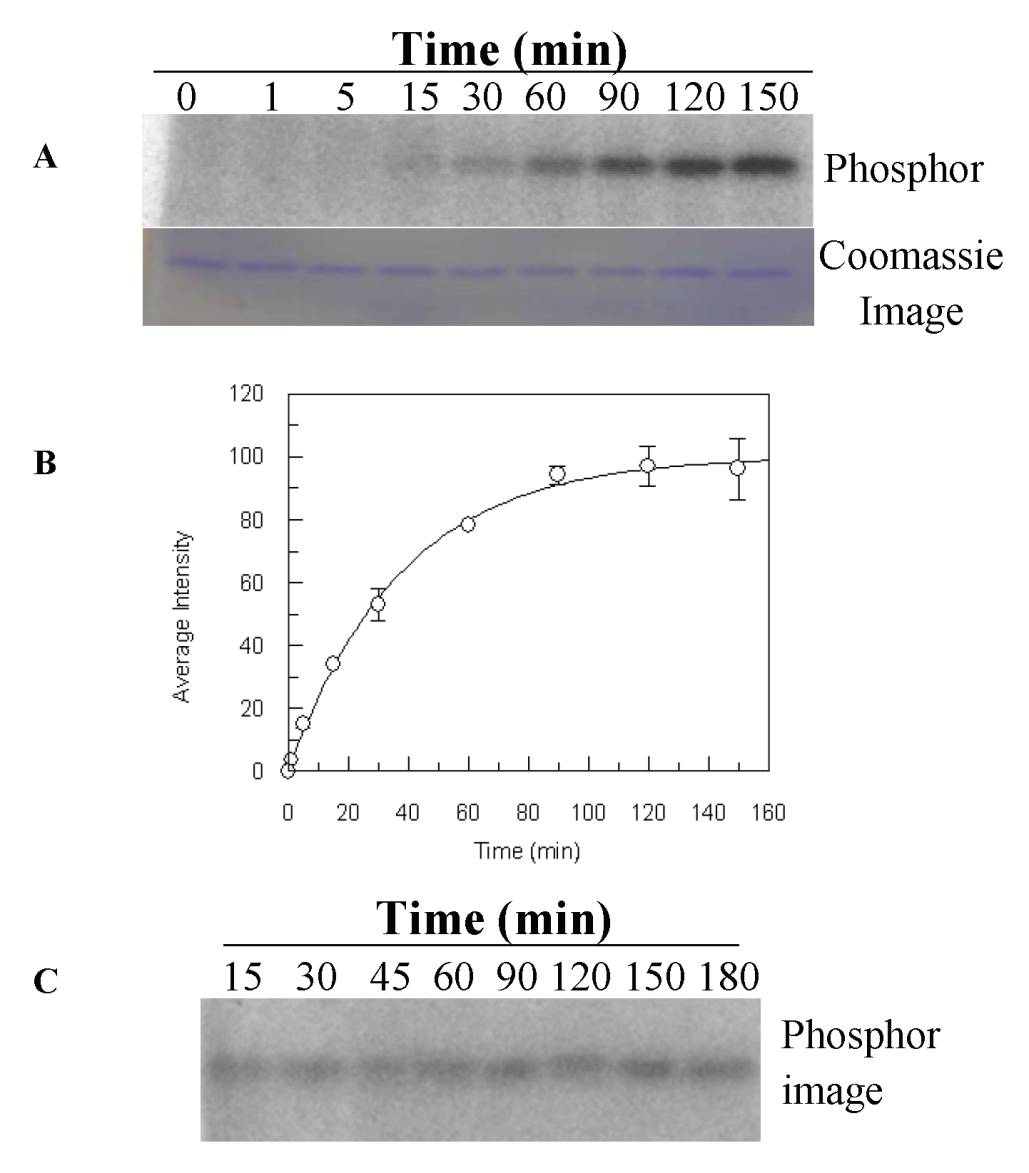
The autokinase activity of His-LytS. ( **A**) His-LytS at 5 µM was incubated with [γ- ^32^P]-ATP (100 µM) in PB at room temperature. Reaction was quenched at different time intervals and samples were analyzed by 15% SDS-PAGE. ( **B**) Progress curve of His-LytS autophosphorylation. The quantified band intensities of phosphorylation were plotted against time. The data were fitted using Origin software to pseudo first order equation (1) to calculate the rate constant. Errors are the standard deviation from two independent trials ( Dataset 1). ( **C**) Stability of the phosphorylated His-LytS species. His-LytS at 5 µM was phosphorylated for 90 min in PB buffer (50 mM Tris, pH 7.4, 5 mM MgCl _2_) at room temperature. Excessive ATP was removed by desalting and stability was monitored over 3 hours at different time intervals. Samples were analyzed by 15% SDS-PAGE. All gels were exposed to phosphor-screen (GE Healthcare) overnight and imaged using a Typhoon Trio ^+^ imager (GE Healthcare). The gel in panel ( **A**) was also stained with Coomassie blue to view the protein bands.

We monitored the level of autophosphorylation of His-LytS at different time intervals and at different ATP concentrations ( Figure 2, Dataset 1). (The preliminary data were partially published as part of a poster presentation in ASBMB in April 2013 ^29^.) The pseudo first-order rate constant of the autokinase activity of His-LytS was determined to be 0.030 ± 0.001 min ^-1^. The autophosphorylation rate constant of LytS is smaller compared to the *S. aureus* cell wall damage sensing HK VraS (0.07 min ^-1^) ^22^, *Enterococci faecium* vancomycin resistance factor HK VanS of (0.17 min ^-1^) ^30^ or *S. aureus* essential HK WalK (0.36 min ^-1^) ^31^. However, it is similar to the autophosphorylation rate constants for other HKs such as *E. coli* nitrate sensing HK NarQ (0.014 min ^-1^) ^32^, and *E. coli* citrate sensing HK DcuS (0.043 min ^-1^) ^33^. The binding affinity of LytS for ATP ( *K*_s_ = 7.9 ± 0.6 µM) is higher in comparison to other kinase such as, VanS ( *K*_m_^ATP^ = 620 ± 42 µM) ^30^, VraS ( *K*_m_^ATP^ = 230 ± 42 µM (Belcheva *et al.* unpublished data)) and WalK ( *K*_m_^ATP^ = 130 µM) ^31^.

### Phosphorylation of LytR

**Figure 3.**
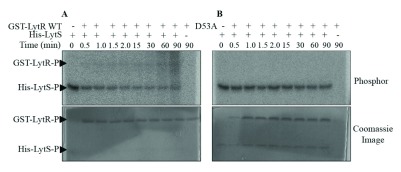
The phosphotransfer between His-LytS and GST-LytR or GST-LytRAsp53Ala variant. ( **A**) Phosphorylated His-LytS at 4 µM was incubated with GST-LytR at 10 µM in PB at room temperature. The reaction was quenched at various time intervals and samples were analyzed by 15% SDS-PAGE. ( **B**) Similar reaction as shown in ( **A**), performed with GST-LytRAsp53Ala. The reaction was quenched at various time intervals and samples were analyzed by 15% SDS-PAGE. Gels were exposed to phosphor screen (GE Healthcare) overnight and imaged (top gels) using a Typhoon Trio ^+^ imager (GE Healthcare) followed with Coomassie blue staining (bottom gels).

We investigated phosphorylation of LytR through its cognate HK, LytS, and the small molecule phosphate donor, acetyl phosphate. Our efforts to investigate the phosphotransfer between His-LytS and LytR were hampered by the fact that the molecular masses of His-LytS and LytR were similar and this affected their separation by SDS-PAGE. To remove this obstacle, LytR was fused to GST which increased the molecular mass of LytR by 26 kD. When P ^32^-labeled His-LytS was incubated with GST-LytR, we observed a time-dependent reduction in signal from P ^32^-labeled His-LytS, which was associated with an increase in P ^32^-labeling of GST-LytR ( Figure 3A). The observed rate constant for the phosphotransfer process is 0.3 ± 0.1 min ^-1^. When incubation of P ^32^-labeled His-LytS was done in the presence of GST-LytRAsp55Asn, no reduction in signal from phosphorylated His-LytS was observed ( Figure 3B). These experiments show that LytS is capable of phosphorylating LytR for as long as the phosphorylation site in LytR is available.

**Figure 4.**
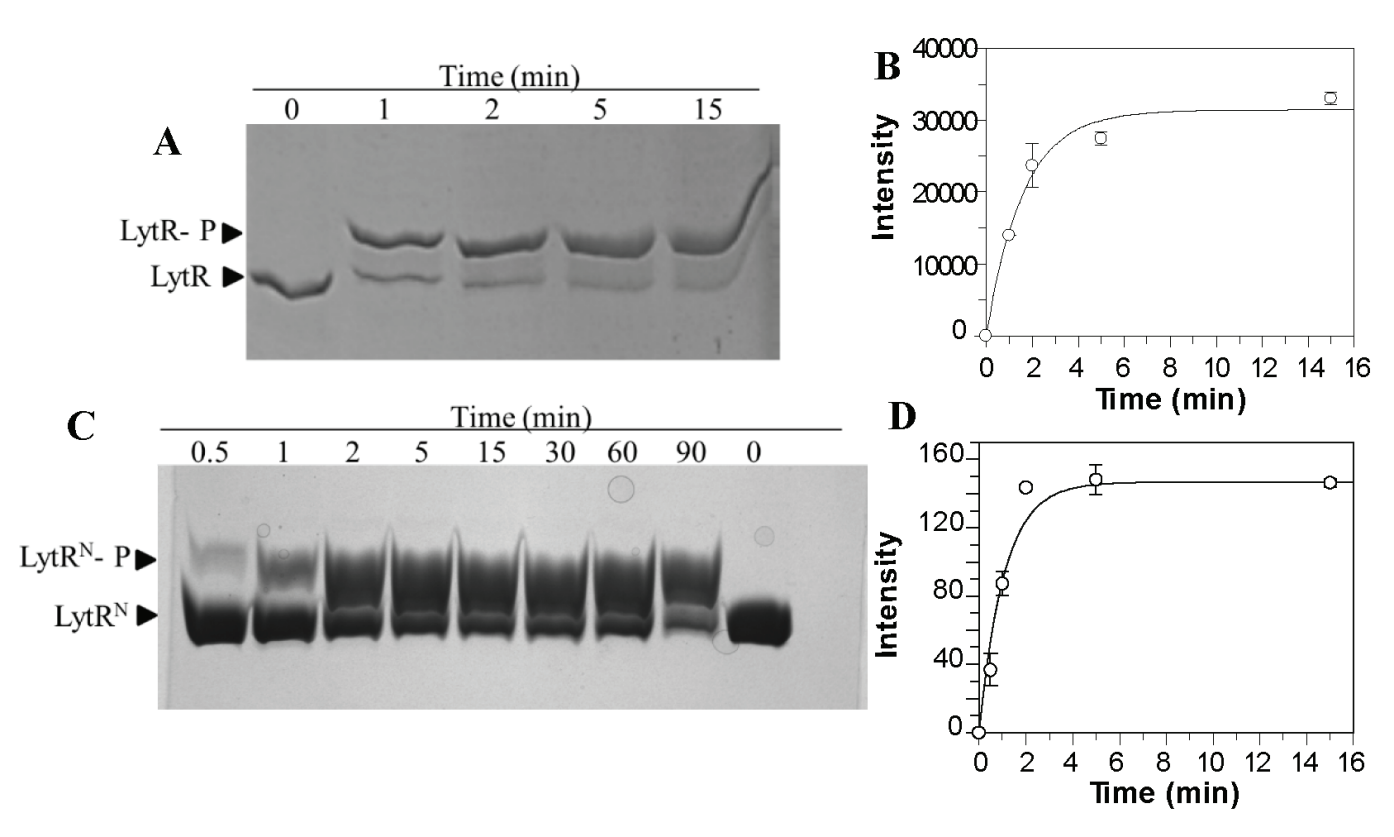
Phosphorylation of LytR and LytR ^N^ by acetyl phosphate. ( **A**) LytR at 10 µM was phosphorylated by acetyl phosphate (50 mM) in PB for 1 h at 37 ^o^C. ( **B**) Quantitative analysis of the data using NIH ImageJ ( Dataset 2) The data were fitted using Origin software to pseudo first order equation (1) to calculate the rate constant. Errors are the standard deviation from two independent trials. ( **C**) LytR ^N^ at 20 µM was phosphorylated by acetyl phosphate (50 mM) in PB buffer at 37 ^o^C and reactions were quenched at various time intervals. The phosphorylated proteins were separated from the unphosphorylated protein by 15% Phos-tag gel. The gels were stained with Coomassie blue to view the protein bands. ( **D**) Quantitative analysis of the data using NIH ImageJ. The data were fitted using Origin software to pseudo first order equation (1) to calculate the rate constant. Errors are the standard deviation from two independent trials ( Dataset 3).

Incubation of LytR with a small molecule phosphate donor such as acetyl phosphate resulted in rapid phosphorylation of LytR ( Figure 4A, Dataset 2). The observed phosphorylation rate constant for LytR was 0.6 ± 0.1 min ^-1^. This rate is about 30-fold faster than phosphorylation of VraR by acetyl phosphate (0.022 min ^-1^) ^22^, or MtrA (0.014 min ^-1^) and PrrA (0.028 min ^-1^), 10-fold faster than DrrD (0.10 min ^-1^), and comparable to PhoB (0.45 min ^-1^) ^34^. In the case of the stand-alone receiver domain, LytR ^N^, the observed phosphorylation rate constant was 0.9 ± 0.2 min ^-1^ ( Figure 4B). The higher phosphorylation rate constant measured for the LytR ^N^ (1.5-fold compared to LytR) is another indication that the effector domain may perturb the receiver domain and very likely it does so through an interdomain interaction. Barbieri *et al.* recently reported a correlation between a higher phosphorylation rate constant for the receiver domains compared to the full-length RRs in cases where domains in RRs were engaged in interdomain interactions ^34^.

**Figure 5.**
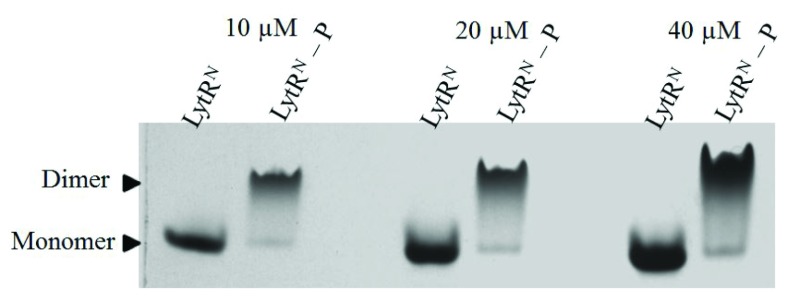
The effect of phosphorylation on the oligomerization states of LytR ^N^. LytR ^N^ and LytR ^N^–P at 10 µM, 20 µM and 40 µM were analyzed by 15% native-PAGE and stained with Coomassie blue.

Investigation of the oligomerization state of LytR was not possible through native-PAGE (Tris-Glycine, pH 8.3) as the protein was not resolved under this buffer condition. Instead, we analyzed the oligomerization state of the phosphorylated LytR ^N^, which resolved well in native-PAGE. These experiments showed that phosphorylation of LytR ^N^ led to dimerization ( Figure 5). These results can be used to predict the oligomerization state of phosphorylated LytR and it is very likely this protein dimerizes upon phosphorylation, and it does so at the receiver domain.

**Figure 6.**
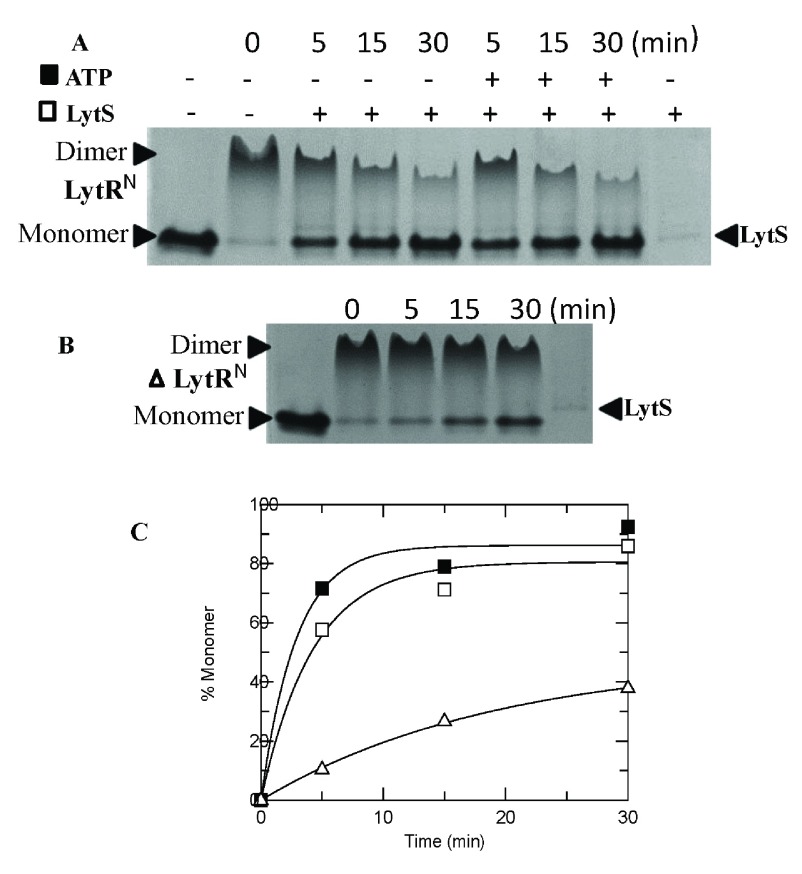
The phosphatase activity of LytS and autophosphatase activity of LytR ^N^. ( **A**) LytS at 5 µM was incubated with LytR ^N^-P at 80 µM at different time intervals, in the absence or presence of 200 µM ATP, at 37°C. ( **B**) The stability of phosphorylated LytR ^N^-P at 37°C. ( **C**) Time-dependence of the phosphatase activity of LytS in the absence of ATP (empty squares) or presence of ATP (solid squares) and autophosphatase activity of LytR (empty triangles). The data points were taken from the analyses of panels A and B using NIH ImageJ ( Dataset 4).

The propensity of LytR ^N^ to dimerize upon phosphorylation was used to monitor the phosphatase activity of LytS; dephosphorylation of LytR ^N^ will lead to disintegration of the dimer which can readily be monitored in a native-PAGE system. Indeed, incubation of the phosphorylated LytR ^N^ with LytS led to conversion of the dimer to monomer species indicating that LytS has phosphatase activity ( Figure 6A, Dataset 4). Interestingly, the phosphatase activity of LytS was more prominent in the presence of ATP similar to the observations made with *E. coli* osmosensor HK EnvZ; this phenomenon was proposed to be due to the allosteric effect that binding of ATP to CA domain had on the phosphatase activity of the DHp domain of EnvZ ^35^.

LytR ^N^ underwent slow dephosphorylation at 37°C ( Figure 6B, Dataset 4). This is an indication that LytR has autophosphatase activity. However, the rate of auto-dephosphorylation of LytR is about 10% of the dephosphorylation rate by LytS ( Figure 6C) hence, it may not be relevant *in vivo*.

Raw data for the role of acetyl phosphate in bypassing the cell membrane electrical potential sensor LytS Data 1 Autophosphorylation of LytS. Quantification of phosphorylated LytS bands by NIH Image J. Average of two trials.
Data 2 Phosphorylation of LytR by acetyl phosphate. Quantification of phosphorylated LytR bands by NIH ImageJ.
Data 3 Phosphorylation of LytR-N by acetyl phosphate. Quantification of phosphorylated LytR-N bands by NIH ImageJ.
Data 4 Quantification of LytR bands in native-PAGEs (Figure 6A and B) by NIH Image J.
LytR protein was phosphorylated by acetyl phosphate and its dephosphorylation by LytS was monitored by native-PAGE, whereby the phosphorylated dimer LytR becomes monomer after dephosphorylation. The column “corrected” represents the data after correcting for background signal at 0 min, and considering the band labeled “Monomer”, in the absence of LytS and ATP, as 100% unphosphorylated LytR. Click here for additional data file. Copyright: © 2015 Patel K and Golemi-Kotra D 2016 Data associated with the article are available under the terms of the Creative Commons Zero "No rights reserved" data waiver (CC0 1.0 Public domain dedication).

## Discussion

LytSR is involved with sense-response to alterations of the cell electrical membrane potential due to cell membrane perturbations ^2, 3^. Its function is related to regulation of genes controlling cell apoptosis, autolysin activity and biofilm formation ^9, 19^. LytR regulates the *lrgAB operon* ^7^ whereby the *lrg* genes products encode antiholin-like proteins that inhibit murein hydrolysis activity and cell lysis ^9^.

To the present day, molecular details and functionality of the LytSR signal transduction pathway have only been presumed. We undertook *in vitro* characterization of LytS and LytR and probed their involvement in the signal transduction process. Signal transduction processes mediated by TCSs involve two reversible phosphorylation-mediated processes, autophosphorylation of the HK and transfer of its phosphoryl group to the cognate RR. Quite often the role of HK is to control the phosphorylation level of RRs, which in turn regulates the transcriptional activity of RR. It does so by possessing phosphatase activity toward RR, in addition to the kinase activity ^13^. The phosphorylation level of RRs, however, can also be regulated through phosphorylation by small molecule phosphate donors such as acetyl phosphate and the autophosphatase activity of RRs ^26^.

Our study demonstrates that the cytoplasmic domain of LytS has autokinase activity ( *k* = 0.030 ± 0.001 min ^-1^). The LytS phosphorylation rate constant is comparable to other HKs. The unusually low dissociation constant measured for ATP with LytS in comparison to other RRs, such as VraR, WalK, or VanS, suggests that the autophosphorylation efficiency for LytS is high and the kinase is well positioned to participate directly in the signalling process induced by changes in the cytoplasmic membrane electrical potential. The fast phosphotransfer process that we observed between LytS and LytR (0.3 min ^-1^) suggests that any alteration in the cell membrane electrical potential sensed by LytS is efficiently transduced intracellularly.

It is well established that most RRs are also equipped with the ability to catalyze their own phosphorylation, independently of their cognate kinases, using endogenous low molecular weight phosphoryl group donors ^36^. In fact, phosphorylation of RRs by low molecular weight phosphoryl group donors such as acetyl phosphate, carbamyl phosphate or phosphoramide appears to be more common than phosphorylation by non-cognate HKs ^37^. Intracellular concentration of acetyl phosphate ranges from 1 mM to 3 mM *in vivo*, suggesting that this phosphate group donor is available in the cell in similar quantities as ATP ^34, 36, 37^. Herein, we show that LytR undergoes rapid and quantitative phosphorylation by acetyl phosphate ( *k* = 0.6 min ^-1^). Further, phosphorylation of the receiver domain by acetyl phosphate leads to dimerization of this domain demonstrating that phosphorylation-induced activation of LytR involves formation of dimers at the receiver domain. The differences in the thermal denaturation and phosphorylation rates by acetyl phosphate of LyR and LytR ^N^ provide evidence that the effector domain has a destabilizing effect on the receiver domain, which could plausibly be due to an interdomain interaction.

The rapid phosphorylation of LytR by acetyl phosphate observed in our study (about 2-fold faster than phosphorylation by LytS) strongly suggests that this pathway is important *in vivo*. Interestingly, *in vivo* studies have demonstrated presence of two overlapping regulatory networks in regulation of the *lrgAB* operon ^1, 20^. One regulatory network responds to excess glucose metabolism and the other responds to changes in the cell membrane potential ^2^. Induction of *lrgAB* in response to glucose metabolism was shown *in vivo* to rely on LytR ^2^, however, metabolism of excess glucose does not lead to changes in the cytoplasmic membrane electrical potential ^1^, hence it is less likely that signaling occurs through LytS in this case. The fast kinetics of LytR phosphorylation by acetyl phosphate makes this pathway an efficient signaling and regulatory mechanism of *lrgAB* in response to the glucose metabolism. Moreover, the phosphatase activity of LytS toward phosphorylated-LytR, which otherwise is stable during the cell division time, provides the regulatory means to shut-down this pathway when the glucose level in the medium goes down.

**Figure 7.**
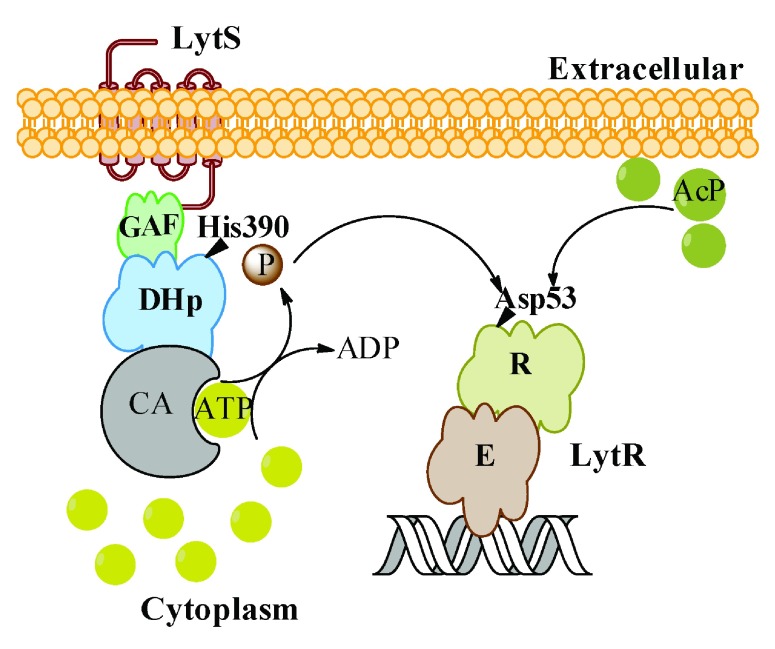
The schematic illustration of the signal transduction by LytSR. Our data support two pathways of activation of LytR: through LytS as a result of perturbation of the membrane electric potential, and through acetyl phosphate as a result of accumulation of acetate during metabolism of excess glucose.

In summary, our study shows that LytSR can participate in two signal transduction pathways through two phosphorylation processes: phosphorylation of LytR by LytS and phosphorylation of LytR by acetyl phosphate ( Figure 7). Further, our findings provide the molecular mechanism for the *in vivo* observation that regulation of *lrgAB* operon is LytR dependent, either in response to excess of glucose metabolism or perturbation of cell membrane electrical potential.

A recent report by Lehman *et al.* showed that phosphorylation of LytR by acetyl phosphate is relevant *in vivo* and it is important for regulation of *lrgAB* operon under high level of glucose ^38^.

## Data availability

The data referenced by this article are under copyright with the following copyright statement: Copyright: © 2016 Patel K and Golemi-Kotra D

Data associated with the article are available under the terms of the Creative Commons Zero "No rights reserved" data waiver (CC0 1.0 Public domain dedication). 

*Figshare:* Raw data for the role of acetyl phosphate in bypassing the cell membrane electrical potential sensor LytS doi: 10.6084/m9.figshare.1339843 ^39^
