# Supplementary material for: Signaling mechanism by the Staphylococcus aureus two-component system LytSR: role of acetyl phosphate in bypassing the cell membrane electrical potential sensor LytS
Source: F1000Res. 2016 Mar 22;4:79. Originally published 2015 Mar 26. [Version 2] doi: 10.12688/f1000research.6213.2 (PMC4830213; doi:10.12688/f1000research.6213.2)
Supplement: Peer review history

# Peer review: Referee response for version 2

**DOI:** 10.5256/f1000research.8994.r13179

**Published:** 2016-04-04

**Authors:** Kenneth W Bayles

**Affiliations:** 1 Department of Pathology and Microbiology, The University of Nebraska Medical Center, Omaha, NE, USA

I have read the revised version of the manuscript by K. Patel K and D. Golemi-Kotra entitled "Signaling mechanism by the Staphylococcus aureus two-component system LytSR: role of acetyl phosphate in bypassing the cell membrane electrical potential sensor LytS” and approve the changes made.

I have read this submission. I believe that I have an appropriate level of expertise to confirm that it is of an acceptable scientific standard.

# Peer review: Referee response for version 2

**DOI:** 10.5256/f1000research.8994.r12979

**Published:** 2016-03-29

**Authors:** Taeok Bae, Won-Sik Yeo

**Affiliations:** 1 Department of Microbiology and Immunology, Indiana University School of Medicine-Northwest, Gary, IN, USA

In this revised manuscript, the authors addressed most of my previous concerns. But, two concerns are still remaining.

**Abstract**

The authors still indicate that LytR regulates cidABC.

**Abstract & Discussion**

In this revised manuscript, the authors say that the phosphotransfer reaction from LytS-P to LytR is fast.

“LytS… can catalyze a fast phosphotransfer reaction, with 50% of its phosphoryl group lost within 1 min of incubation with LytR.”

“The fast phosphotransfer process that we observed between LytS and LytR (0.3 min-1) suggests that…”

I admit that “fast” is a relative terminology and can be interpreted differently, depending on how you see it. In addition, the reaction condition is rather artificial in that variant proteins (i.e., cytoplasmic domain of LytS and GST-fusion form of LytR) were used, and the results might not reflect what is happening in the bacterial membrane. Nonetheless, I still think the presented data do not support the notion of “fast phosphotransfer” based on the following reason.

As the author indicated, the rate constant of phosphotransfer reaction was calculated from “dephosphorylation of LytS-P”, not from “phosphorylation of LytR. If all phosphoryl groups detached from LytS-P are transferred to LytR, that would not matter. However, according to the results in Fig. 3A, that seems not to be the case. In the reaction, the LytR concentration is 2.5 times higher than that of LytS (10 uM LytR vs 4 uM LytS). According to the authors, 50% of 32P signals were lost from LytS-P within 1 min. However, at 1 min, the 32P signal of LytR-P is barely visible, indicating that most of the phosphoryl groups detached from LytS-P were NOT transferred to LytR. It might be argued that the low 32P signal of LytR-P is due to the phosphatase activity of LytS. In this case, we can expect a fast disappearance of LytS-P due to the futile phosphorylation/dephosphorylation of LytR. However, as Fig. 3A shows, LytS-P is very stable, and a significant amount of LytS-P is still seen at 90 min, even in the presence of 2.5 times excess LytR. If majority of phosphor-transfer occurs within 1 min, as the authors indicated in their response, we should see a saturation of LytR-P at 1 min. But, Fig. 3A shows that the LytR-P increases gradually, and its level is highest at 90 min. 

So, I recommend the authors to draw a graph for appearance of LytR-P and disappearance of LytS-P like the one in Fig. 4, which might help to clear up this issue.

We have read this submission. We believe that we have an appropriate level of expertise to confirm that it is of an acceptable scientific standard.

# Peer review: Referee response for version 1

**DOI:** 10.5256/f1000research.6666.r12502

**Published:** 2016-03-07

**Authors:** Kenneth W Bayles

**Affiliations:** 1 Department of Pathology and Microbiology, The University of Nebraska Medical Center, Omaha, NE, USA

The authors of this paper sought to further characterize the signal mechanism of the *Staphylococcus aureus* LytSR two-component system. Previous studies have identified two signals that LytSR responds to: dissipation of membrane potential and excess glucose. Using purified protein, the authors characterize the kinetics of the autophosphorylation of LytS and the phosphorylation of LytR by LytS and acetyl-phosphate. The findings include a higher first-order rate constant for the phosphorylation of LytR by acetyl-phosphate as compared to its cognate kinase, LytS. In addition, the first-order rate constant of LytS autophosphorylation indicates a slow reaction as compared to other kinases. It was also demonstrated that LytR forms a dimer once phosphorylated, that LytS has phosphatase activity which is much more efficient that the auto-dephosphorylation of LytR.

The results of the paper confirm and add greater insight into the molecular signaling mechanism of LytSR as previously described by Lehman *et al*, 2015. Overall, manuscript is well written and the experiments nicely executed. However, as can be seen from the comments below, the primary concern is that the authors seemed to have completely missed the work of Lehman *et al, *2015, which describes much of what is presented in the current manuscript. Thus, the authors should include references to this work as indicated below.

**Abstract**

The closing statement of the abstract seems to be a bit overstated as this is not the “first time” these results have been generated. Indeed, very similar results were presented by Lehman *et al *2015 that acetyl-phosphate is responsible for the LytS-independent phosphorylation of LytR. In fact, the Lehman *et al* paper goes further using *in vivo* studies of mutants affecting acetyl-phosphate levels to show that under conditions of excess glucose acetyl-phosphate contributes to the signaling of LytSR.

**Introduction**

Paragraph 3: The authors state “both operons were also shown to be induced by carbohydrate metabolism and proposed to be regulated through a CidR-dependent signaling pathway ^20^.” This is an inaccurate reference, as *cidABC *has only been shown to be regulated by CidR, whereas *lrgAB *expression is dependent on LytSR.

Paragraph 3: The authors should include a reference to the Lehman *et al *2015 paper, which followed up the work performed by Sharma et al (from the same lab). As stated above, the Lehman *et al *2015 paper demonstrated that acetyl-phosphate does influence LytS-independent phosphorylation of LytR. Thus, the statement that the “molecular basis for this observation remains obscure” (referring to the Sharma *et al.* paper) is misleading.

Paragraph 4: The authors state “Our study shows that LytSR is capable of mediating signaling either through LytS in response to cell membrane electrical potential or through LytR in response to carbohydrate metabolism.” This is overstated as there were no experiments to demonstrate this conclusion.

**Results**

Paragraph 2: It was published by Brunskill and Bayles 1996 that Asp-53 is the likely site of LytR phosphorylation. This observation was confirmed in Lehman *et al*. 2015.

**Discussion**

Paragraph 1: LytR does not regulate the *cidABC *operon as stated.

Paragraphs 2 and 5: See Lehman *et al *2015 which characterized the LytSR signal transduction pathway *in vitro *and *in vivo*.

I have read this submission. I believe that I have an appropriate level of expertise to confirm that it is of an acceptable scientific standard, however I have significant reservations, as outlined above.

## References

[1] LehmanMKBoseJLSharma-KuinkelBKMoormeierDEEndresJLSadykovMRBiswasIBaylesKW: Identification of the amino acids essential for LytSR-mediated signal transduction in Staphylococcus aureus and their roles in biofilm-specific gene expression. *Mol Microbiol*.2015;95(4) : 10.1111/mmi.12902 723-37 10.1111/mmi.12902 25491472PMC4347461

# Response

**Published:** 2016-03-11

**Authors:** Dasantila Golemi-Kotra

**Affiliations:** York University, Toronto, Canada

We are very thankful to the reviewer’s comments and suggestions. We have addressed them point-by-point below:

*The authors of this paper sought to further characterize the signal mechanism of the Staphylococcus aureus LytSR two-component system. Previous studies have identified two signals that LytSR responds to: dissipation of membrane potential and excess glucose. Using purified protein, the authors characterize the kinetics of the autophosphorylation of LytS and the phosphorylation of LytR by LytS and acetyl-phosphate. The findings include a higher first-order rate constant for the phosphorylation of LytR by acetyl-phosphate as compared to its cognate kinase, LytS. In addition, the first-order rate constant of LytS autophosphorylation indicates a slow reaction as compared to other kinases. It was also demonstrated that LytR forms a dimer once phosphorylated, that LytS has phosphatase activity which is much more efficient that the auto-dephosphorylation of LytR.*

*The results of the paper confirm and add greater insight into the molecular signaling mechanism of LytSR as previously described by Lehman et al, 2015. Overall, manuscript is well written and the experiments nicely executed. However, as can be seen from the comments below, the primary concern is that the authors seemed to have completely missed the work of Lehman et al,2015, which describes much of what is presented in the current manuscript. Thus, the authors should include references to this work as indicated below.*

***Abstract***

*The closing statement of the abstract seems to be a bit overstated as this is not the “first time” these results have been generated. Indeed, very similar results were presented by Lehman et al 2015 that acetyl-phosphate is responsible for the LytS-independent phosphorylation of LytR. In fact, the Lehman et al paper goes further using in vivo studies of mutants affecting acetyl-phosphate levels to show that under conditions of excess glucose acetyl-phosphate contributes to the signaling of LytSR.*

To acknowledge the study by Lehman et al. we have included this reference in the new version of the manuscript. We have removed “first time” from abstract as to not divert the attention from the essence of this study.

**Introduction**

*Paragraph 3: The authors state “both operons were also shown to be induced by carbohydrate metabolism and proposed to be regulated through a CidR-dependent signaling pathway ^20^.” This is an inaccurate reference, as cidABC has only been shown to be regulated by CidR, whereas lrgAB expression is dependent on LytSR.*

Paragraph 3 has been modified now to address the reviewers concerns. Of note, it is proposed in several papers that CidR is involved in regulation of both operons. We have cited the work by Patton et al. 2006. The figure 6 in this work indicates the proposed model. We have replaced Ref 20 with Patton et al. report. We have also added the work by Yang et al 2006.

*Paragraph 3: The authors should include a reference to the Lehman et al 2015 paper, which followed up the work performed by Sharma et al (from the same lab). As stated above, the Lehman et al 2015 paper demonstrated that acetyl-phosphate does influence LytS-independent phosphorylation of LytR. Thus, the statement that the “molecular basis for this observation remains obscure” (referring to the Sharma et al. paper) is misleading.*

We have included Lehman et al. paper in the new version of the manuscript.

*Paragraph 4: The authors state “Our study shows that LytSR is capable of mediating signaling either through LytS in response to cell membrane electrical potential or through LytR in response to carbohydrate metabolism.” This is overstated as there were no experiments to demonstrate this conclusion.*

We have been very careful with the use of the language here. We wrote that out study shows that “LytSR **is capable of** mediating signaling….”. Indeed, autophosphorylation activity of kinase LytS, indicates that LytS is capable of participating directly (with no need for accessary) in signal transduction, and fast phosphorylation of LytR by acetyl phosphate indicates that LytR is capable of participating in signal transduction independently of LytS.

***Results***

*Paragraph 2: It was published by Brunskill and Bayles 1996 that Asp-53 is the likely site of LytR phosphorylation. This observation was confirmed in Lehman et al. 2015.*

Because the phosphorylation site in all response regulators is conserved, the purpose of mutating Asp53 to Ala in this study was to confirm that signal can be transduced from LytS to LytR as an act of phosphotransfer between LytS and LytR. Kindly refer to Figure 3.

***Discussion***

*Paragraph 1: LytR does not regulate the cidABC operon as stated.*

We agree. We have removed this statement.

*Paragraphs 2 and 5: See Lehman et al 2015 which characterized the LytSR signal transduction pathway in vitro and in vivo.*

We have recognized the work my Lehman et al. in the new version of the manuscript.

# Peer review: Referee response for version 1

**DOI:** 10.5256/f1000research.6666.r12500

**Published:** 2016-02-24

**Authors:** Taeok Bae

**Affiliations:** 1 Department of Microbiology and Immunology, Indiana University School of Medicine-Northwest, Gary, IN, USA

In Staphylococcus aureus, transcription of the lrgAB operon is positively regulated by the LytSR two component system.  Previous studies demonstrated that LytS, the sensor histidine kinase, is activated by two different kinds of signals: the loss of membrane potential and glucose/acetic acid. In this paper, using purified proteins, the authors assessed the enzymatic activities of LytS and phosphorylation of LytR by LytS and acetyl phosphate. The autophosphorylation of LytS was rather slow, and the LytS-P was stable. The phosphotransfer from LytS-P to LytR was very slow while the LytR phosphorylation by acetyl phosphate was very efficient.  When phosphorylated, the receptor domain of LytR formed a dimer. The phosphatase activity of LytS was also demonstrated. Based on these results, the authors concluded that acetyl phosphate is the signal transducer from the glucose signal.

This manuscript is well-written and, overall, experiments are properly conducted. However, I have concerns on the interpretation of the experiment results. Those concerns are listed below.

**Abstract**

**“Herein, we show that LytS has autokinase activity and can catalyze a fast phosphotransfer reaction, with 50% of its phosphoryl group lost within 1 minute of incubation with LytR.”**

: Indeed, in Fig. 3, the His-LytS-P signal at 1 min was significantly reduced as compared with that at 0 min.  However, the reduction was not accompanied by increase of GST-LytR-P, indicating that the disappeared phosphoryl group was not transferred to GST-LytR. In addition, from 0.5 min to 90 min, the decrease of His-LytS-P signal was very slow. Therefore, the phosphotransfer reaction appears very slow in the condition employed.

**Introduction**

3rd paragraph: The references 20 and 21 do NOT say that CidR is activated by acetyl phosphate.  Ref 20 shows that the acetate (glucose) activates the transcription of cidABC operon through CidR. Ref 21 reports that CidC is a pyruvate oxidase.

The last paragraph:  The in vitro phosphorylation of LytR by acetyl phosphate does not guarantee that acetyl phosphate phosphorylates LytR in vivo.  To my understanding, acetyl phosphate can phosphorylate many response regulators whose phosphorylation is not affected by carbohydrate metabolism.

**Materials and methods**

2nd paragraph, 4th line from bottom: … for Sick Kids, Toronto, Canada. -> ..for Sick Kids, Toronto, Canada).

Page 5, 1st paragraph, 2nd line from bottom:  delete “for” after 25C.

**Results**

Page 7, 1st sentence:  “cognate” might be a better word than “conjugated”.

Page 8, Figure 3: The results, in particular GST-LytR-P signals, are not clear. Nonetheless, the phosphotransfer from LytS-P to LytR is very slow: even at 90 min, a significant amount of His-LytS-P still remains.  The authors reported that the observed rate constant for the reaction is 0.3 min ^-1^. Although I am not an expert in biochemistry, the rate constant seems too high for the slow reaction. In addition, I wonder how the rate constant was calculated:  was it based on the phosphorylation of GST-LytR or dephosphorylation of His-LytS-P?

Page 8, the last sentence: (Figure 4B) -> (Figure 4C & D).

Page 8 & Figure 4:  The observed phosphorylation rate constant for LytR was 0.6 min ^-1^ while it was 0.9 min ^-1^ for LytRN. However, Fig. 4A shows that a majority of LytR was phosphorylated at 1 min while less than half of LytRN was phosphorylated at the time point (Fig. 4C). I understand that two different concentrations (10 uM for LytR and 20 uM for LytRN) were used. But still, to me, the LytR seems to be phosphorylated faster than LytRN.

Page 9, last sentence and Page 10, the first sentence: The authors say **“the phosphatase activity of LytS was more prominent in the presence of ATP..”**. However, to me, the dephosphorylation rates are very similar, regardless of ATP (Fig. 6A).  I wonder whether the difference shown in Fig. 6C at 5 min is statistically significant.

**Discussion**

Page 10 (right column), top sentence:  The authors say **“The fast phosphotransfer process that we observed between LytS and LytR (0.3 min ^-1^) suggests that any alteration in the cell membrane electrical potential sensed by LytS is efficiently transduced intracellularly.” **In my view, the phosphotransfer process is rather slow, and the rate constant (0.3 min ^-1^) might be miscalculated. If I understand the rate constant correctly, 30% of LytR would be phosphorylated within 1 min (or 30% of LytS-P will be dephosphorylated within 1 min?). Nonetheless, Fig. 3A shows that either reaction does not proceed that fast.

Page 10 (right column), the second paragraph from bottom: The authors say **“The rapid phosphorylation of LytR by acetyl phosphate observed in our study (about 2-fold faster than phosphorylation by LytS) strongly suggests that this pathway is important in vivo.” **Although I also think it is likely, the in vitro phosphorylation of LytR by acetyl phosphate cannot serve as a definitive evidence for the in vivo phosphorylation of LytR by acetyl phosphate.  To provide direct evidence, the authors can grow wild type and the mutants of pta (phosphate acetyltransferase) and ackA (acetate kinase) in the presence of glucose; then they can compare the transcript levels of the lrgAB operon.  Since Pta synthesizes acetyl phosphate, no acetyl phosphate will be present in the pta mutant. On the other hand, ackA is converting acetyl phosphate into ATP; therefore, in the ackA mutant, the level of acetyl phosphate will be higher than that in wild type.  If acetyl phosphate is indeed the in vivo mediator of the glucose signal, the transcript level of lrgAB will be lower in the pta mutant while higher in the ackA mutant,as compared with wild type cells.

Page 10 (right column), the second paragraph from bottom: The authors used the reference 20 to introduce two different regulation mechanism of the lrgAB operon. However, the reference 20 is about the regulation of cidABC operon by CidR. I think the reference 1 is more appropriate.

Fig. 7. The model.

To me, the following model fits better to the data presented in the paper.

1. No glucose (nor acetate), full membrane potential

: In this condition, LytS will have a net phosphatase activity due to slow autokinase activity (Fig. 2A), very inefficient phosphotransferase activity (Fig. 3A), and relatively higher phosphatase activity (Fig. 6), resulting in a low expression of lrgAB.

2. No glucose, loss of membrane potential (e.g., gramicidin, CCCP etc)

: The loss of membrane potential will activate the kinase activity of LytS, converting LytS from a phosphatase to kinase. The level of LytR-P will increase, resulting in higher expression of lrgAB.

3. Glucose, full membrane potential

: The efficient phosphorylation of LytR by acetyl phosphate will overcome the phosphatase activity of LytS, resulting in higher expression of lrgAB.

4. Glucose, loss of membrane potential (e.g., gramicidin, CCCP etc)

: LytR will be phosphorylated by both LytS and acetyl phosphate, resulting in maximal expression of lrgAB.

I have read this submission. I believe that I have an appropriate level of expertise to confirm that it is of an acceptable scientific standard, however I have significant reservations, as outlined above.

# Response

**Published:** 2016-03-11

**Authors:** Dasantila Golemi-Kotra

**Affiliations:** York University, Toronto, Canada

**Reviewer 2**

We are very thankful to the reviewer’s comments and suggestions. We have addressed them point-by-point below:

*In Staphylococcus aureus, transcription of the lrgAB operon is positively regulated by the LytSR two component system.  Previous studies demonstrated that LytS, the sensor histidine kinase, is activated by two different kinds of signals: the loss of membrane potential and glucose/acetic acid. In this paper, using purified proteins, the authors assessed the enzymatic activities of LytS and phosphorylation of LytR by LytS and acetyl phosphate. The autophosphorylation of LytS was rather slow, and the LytS-P was stable. The phosphotransfer from LytS-P to LytR was very slow while the LytR phosphorylation by acetyl phosphate was very efficient.  When phosphorylated, the receptor domain of LytR formed a dimer. The phosphatase activity of LytS was also demonstrated. Based on these results, the authors concluded that acetyl phosphate is the signal transducer from the glucose signal.*

*This manuscript is well-written and, overall, experiments are properly conducted. However, I have concerns on the interpretation of the experiment results. Those concerns are listed below.*

***Abstract***

***“Herein, we show that LytS has autokinase activity and can catalyze a fast phosphotransfer reaction, with 50% of its phosphoryl group lost within 1 minute of incubation with LytR.”***

*: Indeed, in Fig. 3, the His-LytS-P signal at 1 min was significantly reduced as compared with that at 0 min. However, the reduction was not accompanied by increase of GST-LytR-P, indicating that the disappeared phosphoryl group was not transferred to GST-LytR. In addition, from 0.5 min to 90 min, the decrease of His-LytS-P signal was very slow. Therefore, the phosphotransfer reaction appears very slow in the condition employed.*

When comparing panels A and B of Figure 3 (top figures), it is clear that majority of phosphor-transfer, as with any other histidine kinase, occurs within 1 min, followed by a slow decrease in phosphor-signal of LytS-P as LytR undergoes dephosphorylation (by LytS) and then, probably, phosphorylated back by LytS-P. We acknowledge that the LytR-P band is faint under the current quality of the picture in this print.

The experimental data in Fig3A, the dephosphorylation of LytS-P, were fitted into first-order rate kinetics to determine the rate constant of the phosphor-transfer. However, a quick view at the phosphor image (although it is only an estimation method) (Fig 3A top) shows that in about 1 min, 50% of the 32P-signal is lost from LytS-P. Dephosphorylation of LytS follows first-order rate kinetics, hence, t1/2 = ln2/k (where t1/2 is the time that is takes for 50% of the reaction to complete, and k is the observed first-order rate constant), then k = ln2/t _1/2_, from which one can readily determine the k value.

**Introduction**

3rd paragraph: The references 20 and 21 do NOT say that CidR is activated by acetyl phosphate.  Ref 20 shows that the acetate (glucose) activates the transcription of cidABC operon through CidR. Ref 21 reports that CidC is a pyruvate oxidase.

Ref. 21 shows among others that *cidABC* operon is involved in generation of acetate in cell under high content of glucose, and Ref 20 shows that CidR regulates the cidABC operon in response to accumulation of acetate at high concentrations of glucose. It is known that (Ref 35 in the discussion) synthesis of acetyl phosphate from acetate and ATP are reversible processes and the enzymes that catalyse these reactions, are encoded by genes ( *pta* and *ackA*) that are nearly constitutive. In addition, it is known that levels of acetyl phosphate vary with the carbon source in the growth medium (Ref 35). We have made the connection here that the loner response regulator CidR (it is not part of a two-component signal transduction) is activated by acetate through acetyl phosphate. However, we have replaced “acetyl phosphate” with “acetate” as to remain closer to the results in Refs. 20 and 21.

*The last paragraph: The in vitro phosphorylation of LytR by acetyl phosphate does not guarantee that acetyl phosphate phosphorylates LytR in vivo.  To my understanding, acetyl phosphate can phosphorylate many response regulators whose phosphorylation is not affected by carbohydrate metabolism.*

This is a good point. It is true that acetyl phosphate is present in the cell. The notion that it could serve as a phosphorylation agent in the response regulator proteins it may seem to contradict the working of two-component systems. However, the kinetics of phosphorylation of response regulators by their cognate kinases and acetyl phosphate will determine whether phosphorylation by acetyl phosphate is relavent *in vivo*. In the case of LytR, our study shows that phosphorylation by acetyl phosphate is faster than phosphorylation by LytS (and faster than phosphorylation of other response regulator). Hence, it is likely to play a role *in vivo*. A recent paper by Lehman et al shows that our findings have correctly predicted what happens *in vivo*.

***Materials and methods***

*2nd paragraph, 4th line from bottom: … for Sick Kids, Toronto, Canada. -> ..for Sick Kids, Toronto, Canada).*

Corrected.

*Page 5, 1st paragraph, 2nd line from bottom:  delete “for” after 25C.*

Corrected.

***Results***

*Page 7, 1st sentence:  “cognate” might be a better word than “conjugated”.*

Corrected.

*Page 8, Figure 3: The results, in particular GST-LytR-P signals, are not clear. Nonetheless, the phosphotransfer from LytS-P to LytR is very slow: even at 90 min, a significant amount of His-LytS-P still remains.  The authors reported that the observed rate constant for the reaction is 0.3 min ^-1^. Although I am not an expert in biochemistry, the rate constant seems too high for the slow reaction. In addition, I wonder how the rate constant was calculated:  was it based on the phosphorylation of GST-LytR or dephosphorylation of His-LytS-P?*

The rate constant of phosphotransfer from LytS to LytR is 0.3 min-1. It was measured from monitoring the loss of P32-signal from LytS-P in Figure 3A (LytR-P signal is relatively low to be measured accurately). In comparison to the phosphorylation by acetyl phosphate, this process is slower, indeed.

*Page 8, the last sentence: (Figure 4B) -> (Figure 4C & D).*

*Page 8 & Figure 4: The observed phosphorylation rate constant for LytR was 0.6 min ^-1^ while it was 0.9 min ^-1^ for LytRN. However, Fig. 4A shows that a majority of LytR was phosphorylated at 1 min while less than half of LytRN was phosphorylated at the time point (Fig. 4C). I understand that two different concentrations (10 uM for LytR and 20 uM for LytRN) were used. But still, to me, the LytR seems to be phosphorylated faster than LytRN.*

We have shown one representative gel for each case. The data, obtained from quantification of the band intensities are provided in the datasets 2 and 3. These data are grafted in Figs 4B and D. Although from the gel it seems as if that at 0.5 min the signal for LytR-P is higher than LytRN-P, overall the fitting of all the date points shows that for LytRN-P the signal increases faster when considering all the data points.

*Page 9, last sentence and Page 10, the first sentence: The authors say **“the phosphatase activity of LytS was more prominent in the presence of ATP..”**. However, to me, the dephosphorylation rates are very similar, regardless of ATP (Fig. 6A).  I wonder whether the difference shown in Fig. 6C at 5 min is statistically significant.*

To provide objectivity on analysing these data, we have plotted the data in Fig. 6C. To us it was interesting that others have observed similar phenomenon as we did. The data are reproducible (but we only have three different trials to comment on statistically significance). Whether it has any relevance *in vivo*, it needs to be investigated further.

***Discussion***

*Page 10 (right column), top sentence:  The authors say **“The fast phosphotransfer process that we observed between LytS and LytR (0.3 min ^-1^) suggests that any alteration in the cell membrane electrical potential sensed by LytS is efficiently transduced intracellularly.” **In my view, the phosphotransfer process is rather slow, and the rate constant (0.3 min ^-1^) might be miscalculated. If I understand the rate constant correctly, 30% of LytR would be phosphorylated within 1 min (or 30% of LytS-P will be dephosphorylated within 1 min?). Nonetheless, Fig. 3A shows that either reaction does not proceed that fast*.

The experimental data in Fig3A, the dephosphorylation of LytS-P, were fitted into first-order rate kinetics. However, a quick view at the phosphor image shows that in about 1 min, 50% of the 32P-signal is lost from LytS-P. Dephosphorylation of LytS follows first-order rate kinetics, hence, t1/2 = ln2/k (where t1/2 is the time that is takes for 50% of the reaction to complete, and k is the observed first-order rate constant), k = ln2/t _1/2_. One can estimate the value of k from this single data point.

*Page 10 (right column), the second paragraph from bottom: The authors say **“The rapid phosphorylation of LytR by acetyl phosphate observed in our study (about 2-fold faster than phosphorylation by LytS) strongly suggests that this pathway is important in vivo.” **Although I also think it is likely, the in vitro phosphorylation of LytR by acetyl phosphate cannot serve as a definitive evidence for the in vivo phosphorylation of LytR by acetyl phosphate. To provide direct evidence, the authors can grow wild type and the mutants of pta (phosphate acetyltransferase) and ackA (acetate kinase) in the presence of glucose; then they can compare the transcript levels of the lrgAB operon. Since Pta synthesizes acetyl phosphate, no acetyl phosphate will be present in the pta mutant. On the other hand, ackA is converting acetyl phosphate into ATP; therefore, in the ackA mutant, the level of acetyl phosphate will be higher than that in wild type. If acetyl phosphate is indeed the in vivo mediator of the glucose signal, the transcript level of lrgAB will be lower in the pta mutant while higher in the ackA mutant,as compared with wild type cells.*

We agree. The recent work by Lehman et al. have carried out the above experiments (Ref 37 in the new version of manuscript).

*Page 10 (right column), the second paragraph from bottom: The authors used the reference 20 to introduce two different regulation mechanism of the lrgAB operon. However, the reference 20 is about the regulation of cidABC operon by CidR. I think the reference 1 is more appropriate*.

Yes, that is correct.

*Fig. 7. The model.*

*To me, the following model fits better to the data presented in the paper.*

*1. No glucose (nor acetate), full membrane potential*

*: In this condition, LytS will have a net phosphatase activity due to slow autokinase activity (Fig. 2A), very inefficient phosphotransferase activity (Fig. 3A), and relatively higher phosphatase activity (Fig. 6), resulting in a low expression of lrgAB.*

*2. No glucose, loss of membrane potential (e.g., gramicidin, CCCP etc)*

*: The loss of membrane potential will activate the kinase activity of LytS, converting LytS from a phosphatase to kinase. The level of LytR-P will increase, resulting in higher expression of lrgAB.*

*3. Glucose, full membrane potential*

*: The efficient phosphorylation of LytR by acetyl phosphate will overcome the phosphatase activity of LytS, resulting in higher expression of lrgAB.*

*4. Glucose, loss of membrane potential (e.g., gramicidin, CCCP etc)*

*: LytR will be phosphorylated by both LytS and acetyl phosphate, resulting in maximal expression of lrgAB.*

We agree. Ref. 1 study supports the above models, in the addition to the recent work by Lehman et al. (Ref 37 in the new version of the manuscript).
